# *Stemphylium lycopersici* and *Stemphylium solani* improved antioxidant system of soybean under chromate stress

**DOI:** 10.3389/fmicb.2022.1001847

**Published:** 2022-11-03

**Authors:** Husna Husna, Anwar Hussain, Mohib Shah, Muhammad Hamayun, Amjad Iqbal, Muhammad Qadir, Syed Asim, In-Jung Lee

**Affiliations:** ^1^Department of Botany, Abdul Wali Khan University Mardan, Mardan, Khyber Pakhtunkhwa, Pakistan; ^2^Microbial Safety Division, Agro-Food Safety and Crop Protection Department, National Institute of Agricultural Science, Rural Development Administration, Wanju, South Korea; ^3^Department of Food Science and Technology, Abdul Wali Khan University Mardan, Mardan, Khyber Pakhtunkhwa, Pakistan; ^4^Department of Applied Biosciences, Kyungpook National University, Daegu, South Korea

**Keywords:** chromium, soybean, antioxidant system, *Stemphylium lycopersici*, *Stemphylium solani*

## Abstract

Ecologists around the world are giving great attention to the metal pollution of agronomic soil. Recently, several techniques have been employed to remediate heavy metals, but the use of microorganisms is cheap, less time-consuming, and easily available. In the current study, the endophytic strains, Cp1 and Cp2 were isolated from sterilized 1–5 cm long root and leaf segments of *Chlorophytum comosum* using Hagem media. To get pure colonies, the strains were repeatedly cultured on potato dextrose agar (PDA) media. The strains Cp1 and CP2 were identified as *Stemphylium lycopersici* and *Stemphylium solani* based on ITS sequencing and neighbor joining (NJ) method. Both strains showed a growth-promoting potential in soybean seedlings exposed to chromate (Cr) stress. Moreover, *S. lycopersici* and *S. solani* improved the Indole-3-acetic acid (IAA), flavonoids, phenolics, protein, and proline contents, whereas, lowered Salicylic acid (SA) production in the seedlings. The selected endophytic fungal strains also promoted the antioxidant system of soybean seedlings through enhanced production of ascorbic acid oxidase (AAO), catalases (CAT), peroxidase, and free radical scavenging enzymes. Both strains bio-transformed the toxic Cr-VI to less toxic Cr-III in the cultural filtrate as well as host plants. In fact, efficient uptake of Cr and its conversion by the isolated endophytic fungal strains could be used as a viable tool to remediate Cr contamination in agricultural soils.

## Introduction

Heavy metal pollution has spread broadly around the world and the main cause of the pollution is industrialization and urbanization. During the last few decades, the world is heading toward global climate change as well as pollution of soil and air (Buzier et al., 2011). Chromium (Cr) is considered as non-essential element for plants that adversely affect the human health and environment (Kabir et al., 2014). In plants including legumes, exposure to elevated levels of chromium can cause oxidative stress. The oxidative stress further inhibits seed germination and seedling development, reduces root shoot biomass, flower size, crop yield and photosynthesis, and can cause mutagenesis (Oliveira, 2012; Singh et al., 2013; Gill et al., 2015). Besides, the accumulation of Cr also disturbs starch and protein metabolism and nitrogen assimilation (Adrees et al., 2015).

Endophytes are endosymbionts that exhibit beneficial role in the healthy growth of the host plant species (Bibi et al., 2018; Khushdil et al., 2019; Nusrat et al., 2019; Ismail et al., 2021; Raid et al., 2021). Several researchers have pointed that endophytes have a significant impact on the growth and development of the host plant under normal as well as stress conditions (Hamayun et al., 2017; Ikram et al., 2018; Muhammad et al., 2019). The symbiotic relationship of endophytes with their host also counteracts the negative consequences of heavy metals stress (Khan et al., 2015a). The endophytes can also synthesize proteins, exopolysaccharides, organic acids, extracellular enzymes, etc., to decontaminate the soil or to enhance the phyto-remediation ability of the host plants (Redman et al., 2011). Fungal endophytes possess a well-developed ability to chelate metals, which helps the host plants to tolerate HMs present in the soil at several folds (Khan et al., 2015a). Despite the fact that endophytes have role in plant growth promotion, their remediation mechanisms have yet to be explored (Bourdel et al., 2016). So, the use of endophytes to interact and accumulate HMs from polluted area has dual advantages. The endophytes can boost HMs-resistance of their host plant and promote host’s agronomic attributes by secreting plant growth hormones, like GAs and Indole-3-acetic acid (IAA) (Ikram et al., 2018; Qadir et al., 2020a,^2021^, 2022b).

Soybean (*Glycine max* L.) is one of the most commercially valued crops and a significant source of food worldwide. Furthermore, soybean is among the most affordable and abundant sources of both edible oil and protein. It is beneficial to human health in terms of heart disease, certain malignancies, and hypoglycemia (Rebholz et al., 2013; Chen et al., 2018; Fan et al., 2022). The productivity and yield of the soybean crop are significantly affected by Cr contamination in the experimental area. The purpose of this study is, therefore, to explore the role of endophytic fungi [*S. lycopersici* (Cp1) *and S. solani* (Cp2)] isolated from the *C. comosum* in *G. max* seedlings grown under chromate stress. The effects of these isolates on growth attributes, phytohormones, plant metabolites, and metal absorption were observed.

## Materials and methods

### Collection of plant materials

The industrial zone of Islamabad (Latitude and longitude coordinates are: 33.673038, 72.984138) was selected from where the plant *C. comosum* was carefully collected in plastic bags. The collected plant specimens were brought to the Plant-Microbe Interaction (PMI) Lab, Department of Botany, Abdul Wali Khan University, Mardan, for further processing.

### Endophytic fungi isolation

To isolate endophytic fungi, 10 healthy leaves and root segments were randomly selected from the plant and washed in tap water. For surface sterilization, the samples were cut into 1–5 cm long segments. The segments were then dipped in 0.5% sodium hypochlorite for 2 min. The surface sterilant was drained out and the plant segments were further treated with 70% ethanol for 2 min. After draining the ethanol solution, the segments were thoroughly washed with autoclaved distilled water and transferred to the Hagem agar plates. The plates containing segments were incubated for 7 days at 28°C. For the validation of surface sterilization, drops of water from the final wash were also plated on Hegam agar media. Pure isolates were obtained by spreading rhizospheric solution on Hagem medium. The morphologically different and distinct colonies were identified. Inoculum from the morphologically different colonies was individually placed on PDA media. Repeated sub-culturing on PDA was carried out till uniform colonies of a single isolate were obtained.

### Tolerance to chromate-induced stress

The endophytic fungi were inoculated individually in 50 ml of Czapek’s (CZ) broth spiked with various concentrations of Cr^+6^ (i.e., 100, 300, 500, 900, or 1,200 ppm) in the form of potassium chromate (K_2_Cr_2_O_7_) in 250 ml flasks. After spiking, the flasks were plugged with cotton and covered with aluminum foil. The flasks were placed in a shaking incubator for 7 days at 28°C and 120 rpm for growth and development. After the incubation, biomass was separated from the culture filtrate *via* filtration. Weight of the fresh fungal biomass was determined to observe the impact of chromate on the fungal growth. The experiment was repeated thrice under the same condition using the same quality of seeds and growth medium.

### Screening experiment

The isolated strains that were capable to endure chromate stress were selected for the plant growth promotion assays. Aiming the conditions, the plants were grown in hydroponics at two fully expanded leaf stages. The selected fungal isolates were applied in the form of fungal spore suspension at the rate of 10^–8^. The plants were kept in the growth chamber for 14 days. At the end of the experiment, the root, and shoot lengths were recorded.

### Profiling of fungal culture filtrate

Fungal endophytes were inoculated in autoclaved Czapeck media spiked with different concentrations of chromium (i.e., 100, 300, 500, 900, or 1,200 ppm) in 250 ml flasks. After inoculation, the flasks were shifted to the shaking incubator. The incubation was carried out at 150 rpm and 28°C for 5–7 days. The contents of the flasks were centrifuged after completion of the incubation time at 504 × g for 10 min. The collected supernatant was then tested for IAA, Salicylic acid (SA), flavonoids, and phenolics.

### Estimation of IAA and SA in fungal culture supernatant

The fungal culture supernatant (1 ml) was mixed with 2 ml of Salkowski’s reagent (50 ml of 35% perchloric acid, 1 ml of 0.5 M FeCl_3_ solution) and the mixture was allowed to stand for half an hour in the dark at room temperature. The colored complex (pink) was quantified by recording the optical density (OD) of the reaction mixture at 530 nm by using a spectrophotometer (Parkin Elmer double beam spectrophotometer) (Tsavkelova et al., 2007). Pure IAA (1, 10, 100, and 1,000 μg/ml) was used as a standard to draw a calibration curve.

For the estimation of SA, fungal culture supernatant (100 μl) was transferred to a tube and 2.9 ml of freshly prepared iron chloride (0.1%) solution was added to it. A violet color was obtained due to a reaction between Fe^3+^ ions and salicylic acid (Warrier et al., 2013). The OD was noted at 540 nm using Perkin Elmer Lambda double-beam spectrophotometer.

### Estimation of flavonoids and phenolics in fungal culture supernatant

Fungal culture supernatant was carefully collected and stored at 4°C for the quantification of total flavonoid contents and total phenolic contents. The aluminum chloride assay was used to quantify total flavonoid content (TFC) according to the method of El Far and Taie (2009). The mixture comprised of fungal culture supernatant (0.5 ml), 10% aluminum chloride (0.1 ml), 10% potassium acetate (0.1 ml), and 80% methanol (4.8 ml). The mixture was rapidly shaken and kept for incubation at room temperature for half an hour. The OD was recorded at 415 nm against blank. The blank solution was prepared by mixing 0.5 ml of ddH_2_O, 0.1 ml of aluminum chloride (10%), 0.1 ml of potassium acetate (10%), and 4.8 ml of methanol (80%). Quercetin was used as a standard.

The total phenolics were quantified by Folin-Ciocalteau reagent (FCR) method with slight modification (Del Rosario Cappellari et al., 2017). Fungal culture supernatant (2 ml) was mixed with 0.5 ml of FCR and the mixture was left to stand for 5 min. After 5 min, 2 ml of sodium carbonate (20%) solution was added and the mixture was incubated at 25°C for 4 min, followed by heating for 1 min in a boiling water bath. The mixture was cooled to room temperature and the OD was noted at 765 nm against the blank. The blank solution was prepared by mixing Folin-Ciocalteau reagent with distilled water at the ratio of 1:1 (v/v). Gallic acid was used to construct a standard curve.

### Taxonomic characterization

Chromate tolerant plant growth-promoting endophytic fungal isolates were grown in Czapek medium and kept at 120 rpm shaking incubator at 28°C for 7 days. Fungal biomass was obtained by filtering the fungal culture. Biomass of the selected isolates was lyophilized and processed for molecular identification. A previously described cetyltrimethyl ammonium bromide (CTAB) method was used to obtain genomic DNA of the selected isolates (Jakucs et al., 2005). A pair of primers including ITS1 (5′-TCCGTAGGTGAACCTGCGG-3′) and ITS4 (5′-TCCTCCGCTTATTGATATGC-3′) was used for the amplification of internal transcribed spacer (ITS) DNA region of 18 S rRNA gene. The obtained reads were aligned using codon code aligner software (Version 9.0.2) and the consensus sequence was subjected to NCBI BLAST analysis for finding matching sequences.^1^ The most closely matching sequences were retrieved and further processed for getting the exact nomenclature of the isolates. Phylogenetic analysis was carried out using Molecular Evolutionary Genetics Analysis (MEGA) software version 5. Neighbor-joining (NJ) method was used to construct a phylogenetic tree based on the nucleotide sequences in MEGA5 using a bootstrap value of 1,000 replicates.

### Plant growth promotion under heavy metal stress

In order to test the effect of endophyte on the growth and secondary metabolites of soybean seedlings, heavy metal tolerant isolates were selected to conduct the pot experiment in hydroponics. Soybean seeds were acquired from Mingora, Swat’s Agricultural Research Institute. Seeds were surface sterilized with 0.1% HgCl_2_ and washed three times with sterile distilled water. Upon germination, the seedlings (in two expanded leaf stages) were transferred to pots comprising half-strength Hoagland’s solution. Each pot of 450 ml capacity received 400 ml of Hoagland’s solution. All the treatments were repeated three times and each replicate had four plants.

Pots were set in a completely randomized design and the treatments were factorial combinations of two factors: fungal inoculation and Cr concentrations (i.e., 0, 10, and 50 ppm). Inoculation was performed to assess the mitigation capability of Cp1 and Cp2 in pot experiment. The fungal spore suspension was prepared and the spore density was maintained at 10^–8^ spore ml^–1^ using a hemocytometer. The spore suspension (1 ml) was then added to each pot and the pots were placed in a LabTech growth chamber (Model; LGC-5101 G) under 30°C, humidity was not regulated and ranged from 50 to 80%, and photoperiod (14 h-light/10 h-dark) for 15 days. After 15 days, plants (in 6–8 leaf stages) were harvested (the whole plant frozen in liquid nitrogen) and stored at –80°C till growth, and stress-related attributes and root colonization were recorded.

### Determination of phytohormones (IAA and SA)

For the determination of IAA in soybean leaves, 0.2 g plant biomass was crushed in 5 ml of 80% ethanol and the suspension was then spun at 504 × g for 5 min. The IAA in the supernatant (1 ml) was observed as described earlier (Tsavkelova et al., 2007).

To observe the salicylic acid (SA), fresh leaves (100 mg) were extracted in 1 ml of ethanol and centrifuged at 504 × g for 5 min. The SA in the supernatant (100 μl) was measured as described earlier (Warrier et al., 2013).

### Determination of flavonoids and phenolics

Fresh soybean leaves (0.5 g) were crushed in 5 ml of 80% ethanol. The resultant mixture was centrifuged at 504 × g for 5 min. The supernatant was carefully collected and quantified for total flavonoid content (TFC) as mentioned earlier (El Far and Taie, 2009). The total phenolics in the plant extract (2 ml) were quantified by Folin-Ciocalteau reagent (FCR) method as described earlier (Del Rosario Cappellari et al., 2017).

### Determination of proline

The procedure of Bates et al. (1973) was used to quantify the proline contents in the leaves of the soybean. Fresh leaves (0.1 g) were crushed in 4 ml of sulfosalicylic acid solution (3%) and the mixture was incubated at 5°C for 24 h. On the next day, the mixture was centrifuged at 5,600 × g for 10 min. Equal volume of the leaves’ supernatant was mixed with acid ninhydrin reagent (comprised of 1.25 g of ninhydrin in the solution of 30 ml of glacial acetic acid and 20 ml phosphoric acid) and the mixture was boiled at 100°C for an hour. The samples were left to cool at room temperature. Toluene (4 ml) was added to the mixture and the toluene layer containing proline was separated with a separating funnel. The OD of the toluene layer was observed at 520 nm. Toluene without a sample was used as a blank.

### Determination of total proteins

Total protein concentration in samples was determined by Lowry et al. (1951) method. Fresh leaves (100 mg) were crushed in 1 ml of phosphate buffer saline (pH 7.5) solution and the mixture was centrifuged for 10 min at 504 × g. Supernatant (100 μl) was diluted with 900 μl of ddH_2_O and mixed with 1 ml of Biuret reagent [composed of 1% copper sulfate (0.5 ml), 2% sodium potassium tartrate (0.5 ml) and 2% sodium carbonate in 0.1 N of sodium hydroxide (50 ml)]. The mixture was shaken for at least 10 min and then added 100 μl of diluted FCR (1:1) to it before incubation at room temperature for half an hour. The optical density was documented at 650 nm using FCR as a blank. Bovine serum albumin (BSA) was used as a standard.

### Determination of total carbohydrates

The phenol-sulfuric acid method was used for the estimation of total soluble sugar content in fresh leaves of the host plants (Mohammadkhani and Heidari, 2008). The soluble sugars were measured by crushing 0.5 g of fresh leaves in 10 ml of ddH_2_O and centrifuged at 504 × g for 5 min. To 100 μl of supernatant, 1 ml of 80% phenol was added and the mixture was vigorously shaken before incubating it at room temperature for 10 min. Concentrated H_2_SO_4_ (5 ml) was added to the mixture and the contents were allowed to stand for an hour on the bench. Absorbance was finally observed at 485 nm.

### Determination of total lipids

Vanillin-phosphoric acid reagent was used to determine total lipids (Van Handel, 1985). Approximately, 0.2 g of leaf sample was ground in chloroform: methanol (2:1 v/v), and the mixture were transferred to the tubes. The contents of each tube were shaken vigorously, 0.8 ml of 0.73% NaCl was added and let it stand on the bench top at room temperature. After the appearance of three layers, the lower layer containing lipids was separated/collected through the separatory funnel. After separation of lipid layer, 0.1 ml of sulfuric acid was added and the mixture was shaken and then heated at 100°C for 10 min. The sample was then cooled and 2.4 ml of vanillin reagent was added. Pure canola oil was used as a standard and the absorbance was recorded at 490 nm upon the appearance of pink color.

### Antioxidant enzymes activity

Enzymatic activities catalase (CAT), ascorbate acid oxidase (AAO), peroxidase (POD), and DPPH (2,2-diphenyl-1-picrylhydrazyl radical) were analyzed using Perkin-Elmer Lambda 25 double beam UV-spectrophotometer.

Catalase’s activity was assayed as described by Goel and Sheoran (2003) with slight modification. Approximately, 3 ml of the reaction mixture contained 2.6 ml of potassium phosphate buffer (pH 7.0 and 50 mm) and 0.4 ml of H_2_O_2_ (15 mm) and 40 μl enzyme. H_2_O_2_ absorbance decreased after its degradation by the activity of catalase enzyme. The catalase activity was estimated as enzymes unit/g/30 s.

For the quantification of ascorbic acid oxidase activity, Oberbacher and Vines (1963) protocol was followed. The AAO activity was monitored after mixing 50 mM potassium phosphate buffer, 0.5 mM ascorbic acid, 0.1 mM H_2_O_2_, and 100 μl of enzyme extract. The absorbance was noted at 290 nm for every 30 s for a period of 5 min against blank. The enzyme activity was expressed in units of mg^–1^ protein. AAO content was calculated by decrease in OD by 0.05 as one enzyme unit is equivalent to decrease in OD by 0.05 per minute.

Peroxidase activity (POD) was determined in soybean samples using guaiacol as a source of reactive oxygen species (ROS). Reaction mixture consisted of 3 ml of 0.1 M PBS (pH 7.0), 30 μl H_2_O_2_ (12.3 mM), 50 μl of 20 mM guaiacol solution and 0.1 ml plant extract. POD activity in the samples was recorded at 436 nm. Guaiacol was used as a blank (Pütter, 1974).

The %DPPH free-radical scavenging activity in soybean leaves were quantified by using the protocol of Mensor et al. (2001) using 0.004% solution of DPPH as a source of free radical. Changes in absorbance were measured at 517 nm against the ethanol by using UV-spectrophotometer.

### Screening for reactive oxygen species

Scharte et al. (2005) method was followed to monitor the buildup of ROS in soybean leaves treated with Cp1 and Cp2 isolates under Cr stress. For staining, leaves were dipped in 3,3′-diaminobenzidine (1 mg/ml; pH 3.8) and vacuum infiltrated five times (5 min each). The leaves were then incubated for 3 h at room temperature. After incubation, the leaves were put in boiling ethanol (96% v/v) for 20 min in order to get rid of the green background (due to chlorophyll) and to enhance visualization of brown spots produced by DAB with H_2_O_2_. Upon cooling, the tissues were observed under light microscope to visualize the brown spots.

### Root colonization

The ability of endophytes to colonize roots was determined using the root microscopy method (Qadir et al., 2022b). The soybean roots were initially washed with distilled water in order to remove the embedding medium. The roots were then subjected to staining with lactophenol cotton blue reagent (5 g of phenol crystals, 0.025 g of cotton blue, 10 ml of lactic acid, 10 ml glycerol, and 10 ml of distilled water). For staining purposes, the soybean root sections were dipped in the droplet of lactophenol cotton blue reagent for 1 min. The excess stain was removed with distilled water. Mycelium was observed initially with low power (40×) and then under high magnification (100×). The walls of the hyphae can readily be seen against the light blue background (Leck, 1999).

### Analysis of Cr

Chromate levels in plants and culture supernatant of fungal isolates were determined by using the modified Community Bureau of References (BCR) sequential extraction method (Kazi et al., 2005). The sequential extraction process consisted of three steps, the first of which was the extraction of exchangeable fractions. To 0.5 g of air-dried samples, 20 ml of 0.11 M acetic acid (a weak acid) was added and the contents were mixed in shaking incubator for 12–24 h at 25–30°C. The samples were centrifuged at 504 × g and 28°C for 3 min to secure the residue. In the second step, reducible fraction was extracted by adding 20 ml of newly prepared 0.5 M hydroxylamine-hydrochloride to the obtained residue of step one. The pH 1.5 of the mixture was maintained with the nitric acid and the mixture was shaken for 16 h at 25–30°C. Centrifugation was done at 504 × g and 28°C for 3 min to secure the second step residue. In step three, the obtained residue from the Step 2 was treated with 5 ml of 30% H_2_O_2_ and the contents were shaken for 1 h. This step was repeated twice and the pH was adjusted to 2 before drying the sample. To the dried sample, 25 ml of 1 M ammonium acetate was added and the contents were incubated at 28°C for 16 h. Centrifugation was done at 504 × g for 20 min and the residue was washed with ultrapure water to remove any remaining reactants and metals from the residue. Flame atomic absorption spectroscopy (AAnalyst 700, Parkin Elmer, Waltham, MA, USA) was used to estimate the Cr in sample residue.

### Statistical analysis

All the experiments were repeated three times and the data obtained from the factorial experimentation were grouped into two categories, i.e., HMs stressed and endophytic fungi inoculated. Using the SPSS program (IBM SPSS Statistics 21), the analysis of variance (ANOVA) and Duncan’s Multiple Range Test (DMRT) were conducted to establish the significance level (*p* < 0.05) for Cp1 and Cp2 inoculation, heavy metal level, and their interaction. Graph Pad Prism was used to draw the graphs (Version 5.03).

## Results

### Screening the fungal isolates for plant growth promotion under chromate-induced stress

Out of 10 isolated strains, seven strains were capable of tolerating Cr stress up to 1,200 ppm (Supplementary Table 1). From the preliminary screening, the most effective metal-tolerant endophytic fungi were selected for plant growth promotion (Supplementary Figure 1). Among the 7-endophytic fungal isolates, Cp1 and Cp2 significantly (*p* < 0.05) alleviated the Cr stress in soybean (Supplementary Figure 1).

### Identification of fungal isolates Cp1 and Cp2

Growth pattern, texture, morphology of colony, hyphae color, and spores revealed that both Cp1 and Cp2 belonged to *Stemphylium* genus (Supplementary Figure 2). However, based on the 18S rRNA sequence analysis, the isolate Cp1 presented maximum homology (100%) with *Stemphylium lycopersici* and Cp2 showed (92%) homology with *Stemphylium solani*. Isolate Cp1 and Cp2 made a clad with *S. lycopersici* and *S. solani* supported by 96 and 100% bootstrap value in the consensus tree (Supplementary Figure 3). Combine results of phylogenetic analysis and sequence homology suggested that the isolate Cp1 was *S. lycopersici* and Cp2 *S. solani*. The ITS sequence of 18S rDNA sequences was submitted to Genbank database under accession numbers MZ558044 (Cp1) and MZ558047 (Cp2).

### Effect of Cr on IAA and SA production by the selected endophytic strains

The production of IAA by *S. lycopersici* under normal condition was 41.83 μg/ml, which was significantly (*p* < 0.05) inhibited under Cr stress. The concentration of IAA in *S. lycopersici* culture supernatant spiked with 100, 300, 600, 900, or 1,200 ppm of Cr was 20, 18, 18, 19, and 18 μg/ml, respectively (Supplementary Figure 4A). However, a significantly (*p* < 0.05) higher concentration of IAA (88 μg/ml) was observed in the isolate *S. solani* under 100 ppm Cr stress as compared to the control (34 μg/ml). Moreover, *S. solani* released significantly (*p* < 0.05) higher amounts of IAA in the medium supplemented with 300 (71 μg/ml), 600 (75 μg/ml), 900 (75 μg/ml), or 1,200 (67 μg/ml) ppm of Cr in comparison to the 0 ppm Cr (Supplementary Figure 4A).

In terms of SA, both isolates *S. lycopersici* and *S. solani* ably produced it in the culture media, but the concentration of SA decreased significantly (*p* < 0.05) in the presence of Cr (Supplementary Figure 4B). The amount of SA in the culture media of *S. lycopersici* supplemented with 0 ppm of Cr was 130 (μg/ml). Highest amount of SA (2,847 μg/ml) was recorded in the Cr free culture media of *S. solani* (Supplementary Figure 4B). However, in the presence of 1,200 ppm Cr in culture medium, SA concentration declined significantly (*p* < 0.05) to 7 μg/ml in *S. lycopersici* and 263 μg/ml in *S. solani* (Supplementary Figure 4B).

### Effect of Cr on flavonoids and phenolics production by the selected endophytic strains

The results revealed that the flavonoid contents by *S. lycopersici* in the culture supernatant supplemented with 1,200 ppm significantly (*p* < 0.05) increased by 26.56 μg/ml as compared to the flavonoids (8.93 μg/ml) in culture supernatant supplemented within 0 ppm Cr (Supplementary Figure 4B). In comparison to *S. lycopersici*, the strain *S. solani* was far more efficient in producing flavonoid contents (111.31 μg/ml) in the absence of Cr, but a significantly (*p* < 0.05) sharp decline was noticed at elevated concentration of Cr (Supplementary Figure 4C). Similarly, *S. lycopersici* filtrate showed a significant (*p* < 0.05) decrease in the phenolic contents with increasing Cr concentration (100–1,200 ppm) in the culture media. In case of *S. solani*, a significant (*p* < 0.05) decrease in phenolics was noticed at 300 and 600 ppm of Cr, which increased significantly (*p* < 0.05) at 1,200 ppm of Cr (Supplementary Figure 4D).

### Effect of endophytes on the shoot and root lengths of soybean under Cr stress

**FIGURE 1:**
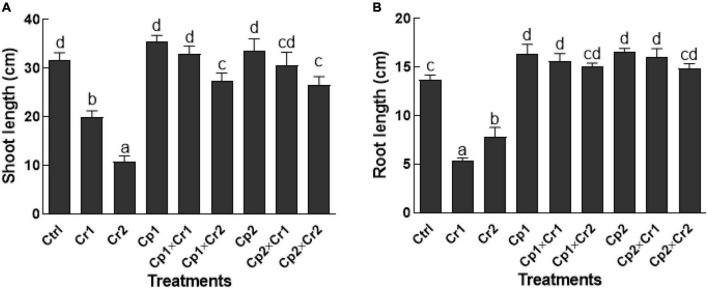
**(A)** Shoot and **(B)** Root length of 15-days-old soybean plants after treatment with selected endophytic fungi exposed to chromate stress. Cp1 = *Stemphylium lycopersici*; Cp2 = *Stemphylium solani*; Ctrl = control; Cr1 = 10 ppm chromium; Cr2 = 50 ppm chromium. Each bar represents mean of triplicated data (*n* = 3) with standard error. Bars that are labeled with different letters are significantly different at *p* < 0.05 computed by Duncan multiple range test.

Exposing plants to 10 or 50 ppm of Cr stress significantly (*p* < 0.05) decrease the root and shoot lengths of the soybean plants as compared to the plants without undergoing Cr stress (Figure 1). Conversely, the effect of chromate stress was significantly (*p* < 0.05) mitigated in the seedlings that were colonized by the selected endophytic fungal strains (Figure 1). The shoot lengths of the fungal-associated soybean plants were not affected (*p* < 0.05), when exposed to 10 ppm of Cr stress. However, at 50 ppm of Cr stress, the reduction in shoot lengths of soybean plants associated with *S. lycopersici* or *S. solani* was reduced by 13.6 and 16.1% (Figure 1A). The root lengths of endophytes-free soybean plant were significantly (*p* < 0.05) reduced by 60.5 and 57% at 10 and 50 ppm of chromate in culture medium. Conversely, chromate exposed endophyte-associated soybean seedlings showed no reduction in root lengths (Figure 1B).

### Effect of endophytes on soluble sugars and proteins of soybean under Cr stress

**FIGURE 2:**
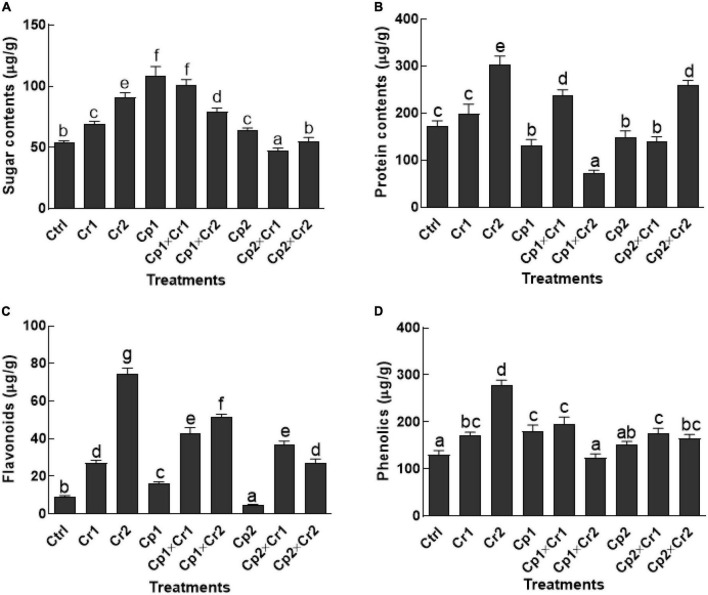
**(A)** Sugars; **(B)** Proteins; **(C)** Flavonoids; **(D)** Phenolics of 15-days-old soybean plants after treatment with endophytes Cp1 and Cp2 exposed to Cr stress. Cp1 = *Stemphylium lycopersici*; Cp2 = *Stemphylium solani*; Ctrl = control; Cr1 = 10 ppm chromium; Cr2 = 50 ppm chromium. Each bar represents mean of triplicated data (*n* = 3) with standard error. Bars that are labeled with different letters are significantly different at *p* < 0.05 computed by Duncan multiple range test.

Exposing soybean seedlings to chromate significantly (*p* < 0.05) increased soluble sugar content as compared to the untreated control seedlings (Figure 2A). Sugar contents were significantly (*p* < 0.05) higher in *S. lycopersici*-associated host plants exposed to 10 and 50 ppm of Cr stress as compared to the control. In case of *S. solani*-associated soybean plants, sugar level declined significantly (*p* < 0.05) at 10 ppm of Cr stress, whereas a non-significant (*p* < 0.05) change was noticed in soybean plants exposed to 50 ppm of Cr stress as compared to the control plants.

In terms of proteins, a significant (*p* < 0.05) increase in the production was noticed in endophyte-free soybean plants under 10 and 50 ppm Cr stress (Figure 2B). The *S. lycopersici*-associated soybean seedlings had significantly (*p* < 0.05) lower level of proteins at 50 ppm of Cr as compared to endophyte-free control plants. Similarly, *S. solani*-associated soybean plants (*p* < 0.05) had significantly (*p* < 0.05) lower level of proteins at 10 ppm as compared to the control plants. The soybean plants associated with *S. lycopersici* and exposed to 10 ppm of chromate had no significant (*p* < 0.05) difference with soybean plants associated with *S. solani* and exposed to 50 ppm of chromate stress. However, in both treatments, the protein contents in soybean plants were significantly higher than their respective controls and endophyte-free control plants (Figure 2B).

### Effect of endophytes on flavonoids and phenolics of soybean under Cr stress

Levels of flavonoids were significantly (*p* < 0.05) enhanced by several folds in Cr stress endophyte-free soybean plants in comparison to the control plants (Figure 2C). Almost 2 to 6-fold increase in flavonoid contents was recorded in soybean seedlings supplemented with 10 and 50 ppm of Cr stress. Soyabean plants associated with *S. lycopersici* exhibited significantly (*p* < 0.05) higher amounts of flavonoids (57.7%) as compared to the control plants under normal conditions (i.e., without exposure to Cr stress). On the contrary, the level of flavonoids was significantly (*p* < 0.05) declined (50.3%) in soyabean plants associated with *S. solani* in comparison to the control plants under normal conditions. Under 50 ppm of Cr stress, the level of flavonoids in endophyte-associated soybean was significantly (*p* < 0.05) lower than the endophyte-free plants (Figure 2C).

Similarly, endophyte-free soybean plants exposed to 10 and 50 ppm Cr stress revealed significantly (*p* < 0.05) higher amounts of phenolics as compared to the plants that didn’t receive Cr stress (Figure 2D). Moreover, the flavonoids contents of the endophyte-free soybeans plants were significant (*p* < 0.05) with *S. lycopersici*-associated plants, but non-significant (*p* < 0.05) with *S. solani*-associated plants under normal conditions. The Association of *S. lycopersici* or *S. solani*, however controlled the production of phenolics significantly (*p* < 0.05) in the soybean plants in comparison to the endophyte-free plants exposed to 50 ppm of Cr stress (Figure 2D).

### Modulation of phytohormones in endophyte-associated soybean under Cr stress

**FIGURE 3:**
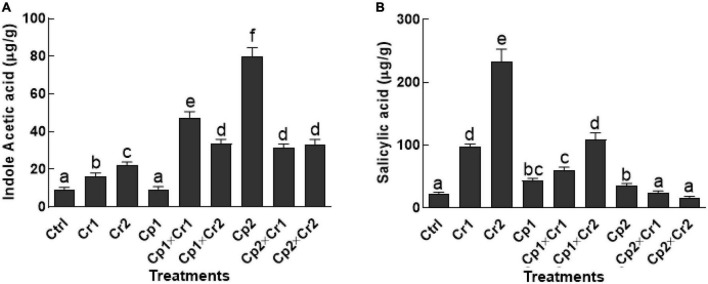
**(A)** Indole-3-acetic acid (IAA); **(B)** Salicylic acid of 15-days-old soybean plants after treatment with endophytes Cp1 and Cp2 exposed to chromate stress. Cp1 = *Stemphylium lycopersici*; Cp2 = *Stemphylium solani*; Ctrl = control; Cr1 = 10 ppm chromium; Cr2 = 50 ppm chromium. Each bar represents mean of triplicated data (*n* = 3) with standard error. Bars that are labeled with different letters are significantly different at *p* < 0.05 computed by Duncan multiple range test.

A dose-dependent rise was noted in the production of IAA contents of the endophyte-free soybean plants, when supplemented with aforementioned levels of Cr (Figure 3A). A 1.5 and 2-fold increase was noted in the endophyte-free soybean plants supplemented with 10 and 50 ppm of Cr. Non-significant (*p* < 0.05) change was detected in *S. lycopersici* inoculated soybean plants, whereas, *S. solani* showed a significant (*p* < 0.05) increase in IAA production under normal conditions. Furthermore, association of *S. lycopersici* and *S. solani* with soybean plants showed a significant (*p* < 0.05) increase in IAA levels as compared to the non-associated seedlings under 10 or 50 ppm of Cr stress (Figure 3A).

A similar trend was recorded for salicylic acid (SA) in endophyte-free soybean plants exposed to Cr stress in comparison to the stress-free plants (Figure 3B). Inoculation of *S. lycopersici* modulated salicylic acid production under Cr stress. A significant (*p* < 0.05) increase in SA was observed, when the *S. lycopersici*-associated soybean plants were supplemented with 10 or 50 ppm of Cr. On the other hand, *S. solani*-associated soybean plants showed an opposite trend, where the SA levels were non-significant in plants that were exposed to 10 or 50 ppm of Cr stress (Figure 3B).

### Effect of endophytes on catalase, ascorbate acid oxidase, and peroxidase activity of soybean under Cr stress

**FIGURE 4:**
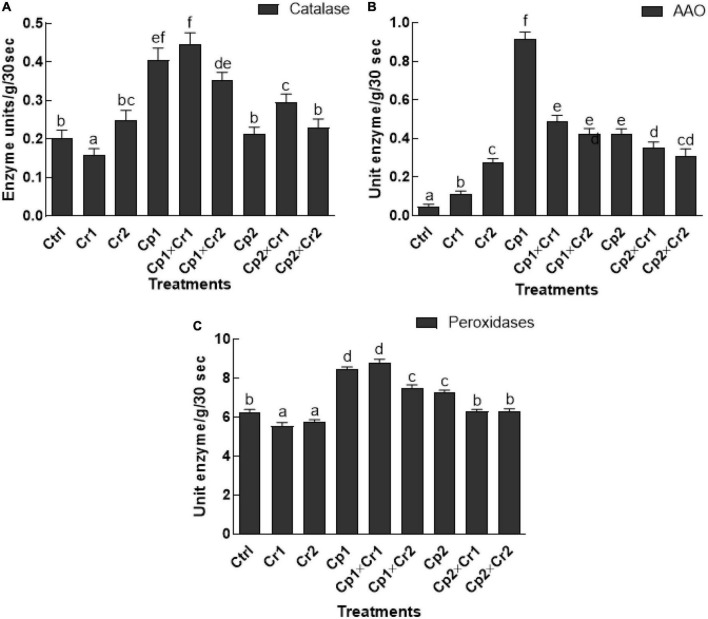
**(A)** Catalase (CAT); **(B)** Ascorbic acid oxidase (AAO); **(C)** Peroxidase. *In vitro* studies on the effect of isolate Cp1 and Cp2 on stress-related enzymes of soybean seedlings exposed to chromate stress. Cp1 = *Stemphylium lycopersici*; Cp2 = *Stemphylium solani*; Ctrl = control; Cr1 = 10 ppm chromium; Cr2 = 50 ppm chromium. Each bar represents mean of triplicated data (*n* = 3) with standard error. Bars that are labeled with different letters are significantly different at *p* < 0.05 computed by Duncan multiple range test.

Soybean leaves exposed to Cr stress showed a significant (*p* < 0.05) decline in catalase activity at 10 ppm of Cr stress, where the activity was increased at 50 ppm of Cr (Figure 4). More interestingly, the soybean plants inoculated with *S. lycopersici* or *S. solani* showed significantly higher CAT activity at 10 ppm of Cr stress as compared to the endophyte-free control plants (Figure 4A). Under stress-free conditions and without endophytic association, the activity of ascorbic acid oxidase (AAO) was significantly (*p* < 0.05) low (Figure 4B). However, the activity of the AAO in soybean seedlings after association with *S. lycopersici* or *S. solani* was significantly (*p* < 0.05) high. Highest activity of AAO (0.91 unit enzyme/g/30 s) was observed in the soybean plants inoculated with *S. lycopersici*, whereas the lowest activity (0.047-unit enzyme/g/30 s) was observed in endophyte-free controls (Figure 4B). The POD activity in endophyte-free soybean plants was significantly (*p* < 0.05) high, when exposed to 10 or 50 ppm Cr stress as compared to the control plants (Figure 4C). Moreover, highest activity of POD was observed in seedlings treated with *S. lycopersici* (8.42 enzyme/g/30 s) and *S. solani* (7.24 enzyme/g/30 s) under normal conditions. However, a significant (*p* < 0.05) decline in POD activity was noticed in *S. lycopersici* associated seedlings under 50 ppm as compared to the associated plants under 10 ppm of Cr stress (Figure 4C).

### Effect of endophytes on 2,2-diphenyl-1-picrylhydrazyl radical activity and proline content of soybean under Cr stress

**FIGURE 5:**
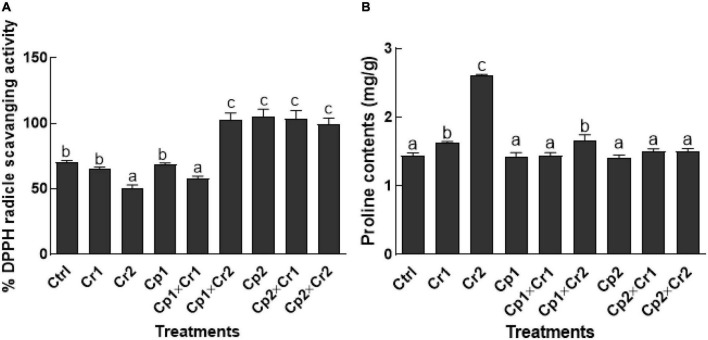
**(A)** 2,2-diphenyl-1-picrylhydrazyl radical (DPPH) radical scavenging activity; **(B)** Proline contents of 15-days-old soybean plants after treatment with endophytes Cp1 and Cp2 exposed to chromate stress. Cp1 = *Stemphylium lycopersici*; Cp2 = *Stemphylium solani*; Ctrl = control; Cr1 = 10 ppm chromium; Cr2 = 50 ppm chromium. Each bar represents mean of triplicated data (*n* = 3) with standard error. Bars that are labeled with different letters are significantly different at *p* < 0.05 computed by Duncan multiple range test.

According to the results, 50 ppm of Cr stress significantly (*p* < 0.05) reduced the DPPH activity in endophyte free soybean plants in comparison to the control (Figure 5A). However, significantly (*p* < 0.05) high DPPH activity was noticed in endophytic fungal (*S. lycopersici* or *S. solani*) associated soybean plants under 50 ppm of Cr stress (Figure 5A). In terms of proline contents, a significant (*p* < 0.05) increase was noticed in endophyte-free soybean plants exposed to elevated levels of Cr (Figure 5B). Co-cultivation of *S. lycopersici* with soybean seedlings didn’t show any significant (*p* < 0.05) change at 10 ppm of Cr stress as compared to the stress-free seedlings. Interestingly, a significant (*p* < 0.05) increase was noted as the *S. lycopersici*-associated plants were supplemented with 50 ppm of Cr. Contrarily, soybean seedlings inoculated with *S. solani* and exposed to 50 ppm of Cr didn’t show any significant (*p* < 0.05) change in the proline contents of the soybean plants (Figure 5B).

### Biotransformation of Cr (VI) and Cr (III) in fungal culture filtrate

**FIGURE 6:**
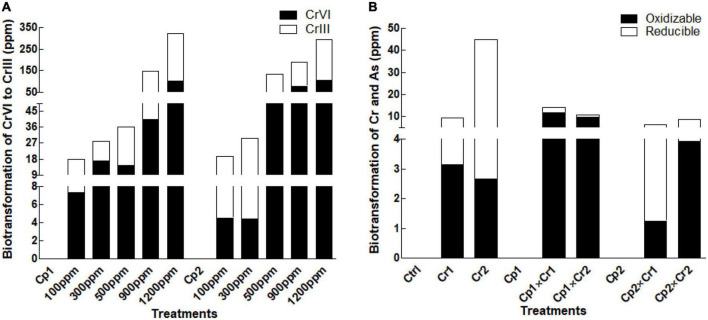
Biotransformation of **(A)** Chromate in fungal culture filtrate **(B)** Chromate in soybean plants inoculated with *Stemphylium lycopersici* and *Stemphylium solani* using the BCR sequential extraction procedure. Each bar represents mean of triplicated data (*n* = 3).

In the current study, the opted isolates *S. lycopersici* and *S. solani* exhibited promising ability to bio-transform the Cr effectively from highly toxic Cr (VI) to less toxic form Cr (III) (Figure 6A). The strain bio-transformed approximately half of the Cr at lower concentration (i.e., 500 ppm). Interestingly, the strain bio-transformed the Cr more effectively when the concentration of the Cr increased in the medium (Figure 6A).

### Biotransformation of Cr in plant biomass

Exposing plant to levels of chromate showed active uptake and biotransformation in the host tissue (Figure 6B). After *S. lycopersici* inoculation, the chromium uptake and their subsequent biotransformation were almost doubled. A contrast results were noted in case of *S. solani* inoculation, where comparatively lower Cr uptake biotransformation was recorded at the supplemented levels of Cr (Figure 6B).

### Reactive oxygen species accumulation

A rise in the level of H_2_O_2_ was noted with the elevation of Cr level from 10 to 50 ppm (Supplementary Figure 5). A substantial quantity of H_2_O_2_ was buildup in the segment of the leaves of soybean seedlings when supplemented with mentioned levels of Cr. With the inoculation of the endophytic fungal isolates *S. lycopersici* and *S. solani*, the Cr stress was alleviated at both concentrations, hence reducing the H_2_O_2_ production and stayed unstained after DAB application (Supplementary Figure 5).

### Root colonization of *Stemphylium lycopersici* and *Stemphylium solani*

Root colonization was detected in the roots after staining with lactophenol cotton blue. The results confirmed the location of endophytic fungal colonization by *S. lycopersici* and *S. solani* strains (Supplementary Figure 6).

## Discussion

Most of the food crops species are prone to heavy metal stress, which spread around the globe due to the industrial revolution and fast urbanization (Buzier et al., 2011). These heavy metals can seize plant growth and intensify ionic imbalance (Ikram et al., 2018). Moreover, the heavy metal chromate is believed to be a non-vital element for plants that harmfully affect the human health and the environment (Kabir et al., 2014). Deposition of Cr in larger quantities inside the crop plant species might inhibit seed propagation, seedling growth, root shoot biomass, flower size, crop yield, and photosynthesis (Singh et al., 2013; Gill et al., 2015). Once hexavalent chromium enters the plant cells via sulfate transporters and gets reduced to Cr(III) by enzymatic and non-enzymatic processes. Through this process, ROS are produced that exert harmful effects on cells by interacting with protein as well as nucleic acid. At a concentration of 3.2 mM, Cr(III) inhibited the germination in *Glycine max, Vigna ratiata*, *Vigna angularis*, and *Lablab purpureis* (Ertani et al., 2017). However, certain plants can tolerate chromate and other heavy metals by a number of mechanisms including precipitation, exclusion, biosorption, reduction, and heavy metal efflux (Cervantes et al., 2001). Being susceptible to environmental stresses the soybean tries to maintain a healthy symbiotic association with surrounding microbes to alleviate the environmental constrains (Vaishnav et al., 2019). In this context, grave attempts are in progress at larger scale to produce genotypes against various stresses through molecular approaches (Ikram et al., 2018). Transfer of useful genes is not only time-demanding, but extremely expensive and laborious. Development of innovative methods, for instance, the application of endophytes to manage stress is a fascinating approach. Cheapness, cleanliness, and rapidity of the method make it a standout approach. Certainly, endophytic fungi have a huge impact on their host plants, inducing their tolerance to biological and abiotic stresses, promoting their growth and the production of various secondary metabolites (Hamayun et al., 2017; Ismail et al., 2020, 2021). In the current study, we used *C. comosum* for the isolation of endophytic fungi and testing the growth-promoting activities of the isolated endophytes in soybean plants. The isolated endophytes significantly (*p* < 0.05) increased the growth parameters (root and shoot length) of soybean plants compared to control plants under normal as well as Cr stress conditions. The endophytic isolates, *S. lycopersici* and *S. solani* also noticed to secret phytohormones (IAA and SA), which helped the soybean plants to achieve normal growth under Cr stress (Qadir et al., 2022b). Increased levels of Cr in the growth medium enabled the *S. lycopersici* and *S. solani* to release optimum amounts of IAA and SA. In fact, controlled production of these phytohormones by the endophytes associated plants under elevated Cr levels can benefit the host plants to withstand the stress (Qadir et al., 2020b,^2021^, 2022b). Evidence from previous studies suggest that role of SA is not limited to its role as defense hormones but it also plays a crucial part in controlling plant growth, germination of seeds, photosynthesis, senescence, and flower/seed formation. However, keeping other parameters constant, an increase in the endogenous level of this hormone is associated with reduced growth (Rivas-San Vicente and Plasencia, 2011). In our study, enhanced level of SA did not impose growth penalty because several other parameters were also modulated as a result of rhizobacteria association in the seedlings. Continuous cropping and climate change threaten plant production via multiple abiotic stresses induced by heavy metals, salinity, ozone, ultraviolet, temperature, and drought (Koo et al., 2020). Intriguingly, SA is not only regulating the resistance to biotic stresses, but also the tolerance to various abiotic stresses (Szalai and Janda, 2007; Khan et al., 2015b). The underlying mechanisms of SA-induced abiotic stress tolerance include (1) accumulation of osmolytes, such as glycinebetaine, proline, soluble sugars and amines, which can help maintain osmotic homeostasis, (2) regulation of mineral nutrition uptake, (3) enhanced ROS scavenging activity, (4) enhanced secondary metabolite production, such as terpenes, phenolics, and compounds with nitrogen (alkaloids, cyanogenic glucosides, non-protein amino acids) and sulfur (glutathione, glucosinolates, phytoalexins, thionins, defensins, and allinin), and (5) regulation of other hormone pathways. Various metabolites, like phenol, proline, and flavonoids were also observed in the culture medium of *S. lycopersici* and *S. solani*. The accumulation and release of metabolites (i.e., phenolics and flavonoids) has been reported in different endophytic fungi, including *Aspergillus* sp. isolated from *Ginkgo biloba*. The release of these metabolites helped the host plants by lowering the accumulation of the ROS under Cr stress (Qiu et al., 2010; Khan et al., 2015a). Antioxidant property of natural products is attributed to the secondary metabolites of the plant kingdom, particularly phenolics and flavonoids has been shown to possess antioxidant activity (Nusrat et al., 2019; Qadir et al., 2020a; Ismail et al., 2021). In our study, we found lower amounts of flavonoids in non-inoculated soybean plants as compared to endophyte treated plants under Cr stress, which suggests the beneficial role of the selected endophytes. It is well-known that the fungal endophytes secrete flavonoids in the host plant tissues to enhance endogenous levels of isoflavones (Qiu et al., 2010). Higher concentrations of flavonoids and antioxidant enzymes have been shown to ease the adverse effect of stress in endophyte-associated *Sesbania sesban* (Abd_Hallah et al., 2015). This effect might be attributed to the scavenging/neutralization of ROS that in turn favor the normal metabolism in the host plant species under stress (Abd_Hallah et al., 2015). Phenolics are known to accumulate in plants under stressful conditions and have beneficial role for plants. Its concentration increases by many folds in traumatic situations as compared to normal conditions (Ismail et al., 2021). We also found higher concentrations of phenolics in the endophytes-associated soybean plants as compared to the control plants under Cr stress. Our findings confirmed those of Ismail et al. (2020), who reported that symbiotic microbes provide resistance to their host plant under stressful conditions. Conversely, lower quantities of proline in the *S. lycopersici* and *S. solani*-associated soybean plants as compared to the free plants were observed under Cr stress. Proline is an osmoprotectant that accumulates inside the plants, when it feels insecure. Proline in fact secure the plant cell wall and structure of proteins, modulating the enzymatic activities, quenching singlet oxygen, and scavenging ROS under severe threat (Kaur and Asthir, 2015). However, low quantities of prolines are produced by plants under normal circumstances or when the plant do not feel high threat (Qadir et al., 2020a). In a study conducted by Abdelaziz et al. (2019) on *Solanum lycopersicum* L. plants under salinity stress, higher amounts of proline were found in *Pyriformis indica* free plants. However, normal proline contents were secreted in the tomato plants inoculated with endophytic *P. indica* that indicated low toxicity of salinity.

To reduce the ROS toxicity, endophytic fungi help the host plants to develop specialized antioxidant system under adverse conditions to cope with it (Ismail et al., 2020). Plants have enzymatic antioxidant defense systems like AAO and CAT that deactivate ROS by degrading H_2_O_2_, hydroxyl radicals, singlet oxygen, and superoxide’s, thus protects plants from the harmful effect of ROS (Ismail et al., 2020). We observed higher CAT activity in Cr stressed and endophytes associated-soybean plants, which means that the endophytes *S. lycopersici* and *S. solani* have provided strength to the plants under stress. Similarly, in another study, a decline in catalase activity under lead-toxic conditions was observed in rice that delayed the lessening of hydrogen peroxide toxicity (Verma and Dubey, 2003).

Endophytic fungi are known to tolerate and detoxify heavy metals through a variety of mechanisms including valence conversion of metal ions, active uptake, extra and intracellular biomineralization (Zahoor et al., 2017; Qadir et al., 2020b,Qadir et al., 2022a). Isolates, *S. lycopersici* and *S. solani* interacts beneficially with the host soybean plants and improved tolerance against Cr in the present study. Although, there is a lack of understanding of the actual mitigation mechanisms used by these microbes, but fungal endophytes are very promising in the bioremediation and restoration of contaminated medium (Deng and Cao, 2017). They not only have the detoxification effect shown here but also have higher resistance to heavy metals (Qadir et al., 2022b).

## Conclusion

Isolates *S. lycopersici* and *S. solani* microbial cells released enough phytohormones and metabolites to cope with the heavy metal stress. Furthermore, *S. lycopersici* and *S. solani* isolate showed promising plant growth-promotion in the presence of HMs. Therefore, such isolates may be of prime importance for developing potential bio inoculant for restoration of Cr-contaminated soil while enhancing the crop productivity with the production of plant growth regulators. In the future we can use these isolates as biofertilizers and bioremediating agents.

## Data availability statement

The original contributions presented in this study are included in the article/Supplementary material, further inquiries can be directed to the corresponding authors.

## Author contributions

HH, MS, MQ, and SA: methodology, experimentation, investigation, validation, data collection, and original draft. AH, MH, and I-JL: conceptualization, supervision, project administration, resources, and funding acquisition. AH, AI, and I-JL: data curation, formal analysis, and writing—reviewing and editing. All authors contributed to the article and approved the submitted version.

## Footnotes

1
http://www.ncbi.nlm.nih.gov/BLAST/
