# Supplementary material for: Stemphylium lycopersici and Stemphylium solani improved antioxidant system of soybean under chromate stress
Source: Front Microbiol. 2022 Nov 3;13:1001847. doi: 10.3389/fmicb.2022.1001847 (PMC9668875; doi:10.3389/fmicb.2022.1001847)
Supplement: Supplementary file 1 [file Data_Sheet_1.docx]

**Supplementary Files**

**Table S1: List of endophytic fungi isolated from *Chlorophytum comosum* and screening from HMs stress**

|  | **Cr stress** | | | | | |
| --- | --- | --- | --- | --- | --- | --- |
|  | 0 | 100ppm | 300ppm | 500ppm | 900ppm | 1200ppm |
| Dgw | +/+ | +/+ | +/+ | +/+ | +/+ | +/+ |
| Yw | +/+ | +/+ | +/+ | +/+ | +/+ | +/+ |
| Bk | +/+ | +/+ | +/+ | +/+ | +/+ | +/+ |
| Bbw | +/+ | +/+ | +/+ | +/+ | +/+ | +/+ |
| Grey | +/+ | **-/-** | -/- | -/- | -/- | -/- |
| B-Grey | +/+ | **-/-** | -/- | -/- | -/- | -/- |
| Cp1 | +/+ | +/+ | +/+ | +/+ | +/+ | +/+ |
| Cp2 | +/+ | +/+ | +/+ | +/+ | +/+ | +/+ |
| Pi | +/+ | +/+ | +/+ | +/+ | +/+ | +/+ |
| Wh | +/+ | **-/-** | -/- | -/- | -/- | -/- |

Screening of fungal endophytes for HM tolerance (+ Fungal growth) (- No fungal growth).


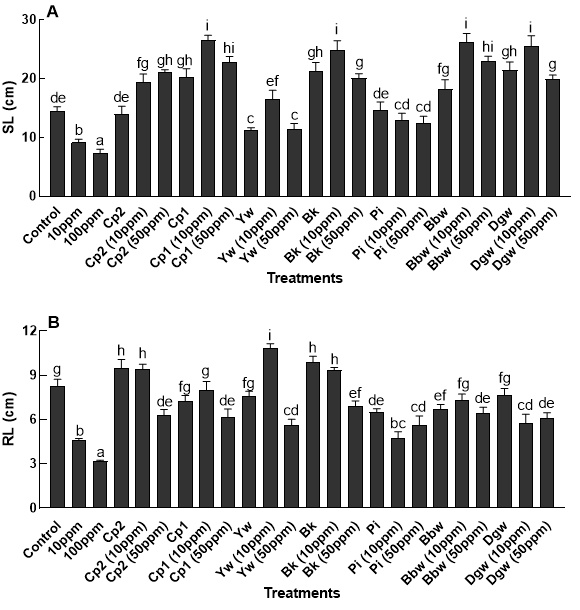


**Figure S1:** Screening the 7 endophytic fungal strains isolated from *Chlorophytum comosum* for plant growth promotion under heavy metal stress on shoot and root length of *Glycine max* L. Each bar represents mean of triplicated data (n=3) with standard error. Bars that are labelled with different letters are significantly different at p < 0.05 computed by Duncan multiple range test.


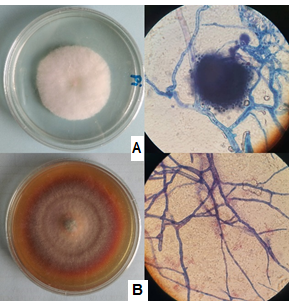


**Figure S2:** Morphological identification of Cp1 (A) and Cp2 (B).


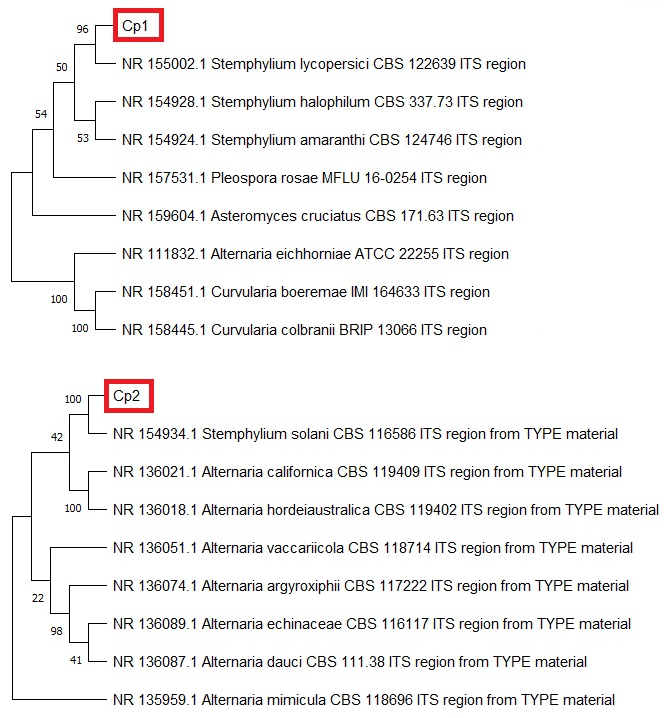


**Figure S3:** Molecular identification of Cp1 and Cp2. Phylogenetic consensus tree construction for the identification of fungal isolate Cp1 and Cp2 using neighbor joining (NJ) method. Bootstrap value confirmed isolate Cp1 as *Stemphylium lycopersici* and Cp2 as *Stemphylium solani*. The ITS sequence of 18S rDNA sequences were submitted to Genbank database under accession numbers MZ558044 (Cp1) and MZ558047 (Cp2).


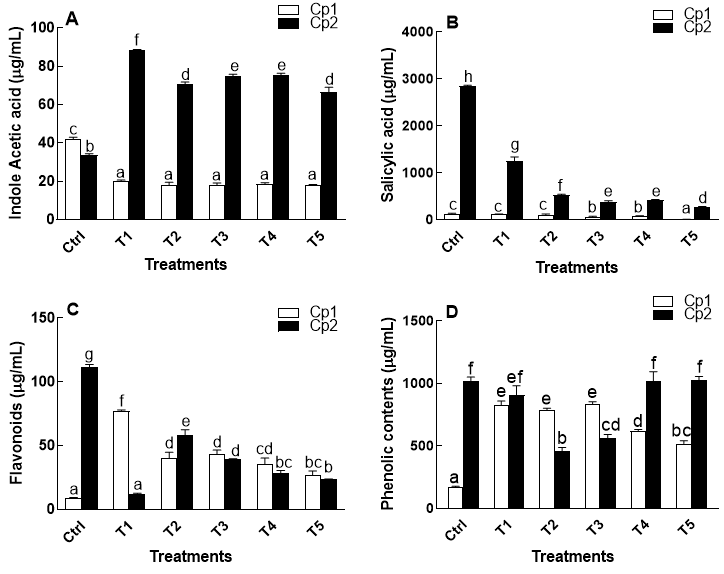


**Figure S4:** Fungal culture filtrate of Cp1 and Cp2 exposed to various concentration of chromate stress. A) IAA; B) Salicylic acid; C) Flavonoids; D) Phenolics. Cp1 = *S. lycopersici*; Cp2 = *S. solani*; Ctrl = control; T1 = 100 ppm chromium; T2 = 300 ppm chromium; T3 = 600 ppm chromium; T4 = 900 ppm chromium; T5 = 1200 ppm chromium. Each bar represents mean of triplicated data (n=3) with standard error. Bars that are labelled with different letters are significantly different at p < 0.05 computed by Duncan multiple range test.


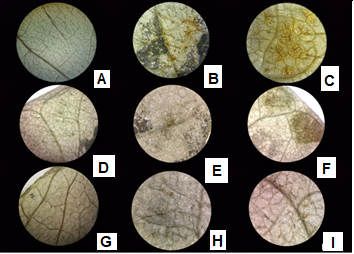


**Figure S5:** ROS accumulation assay with DAB; localized brown areas on the leaves symbolizing ROS accumulation in A) Control; B) Chromate (10ppm); C) Chromate (50ppm); D) Cp1; E) Cp1 (Chromate 10ppm); F) Cp1 (Chromate 50ppm); G) Cp2; H) Cp2 (Chromate 10ppm); I) Cp2 (Chromate 50ppm).


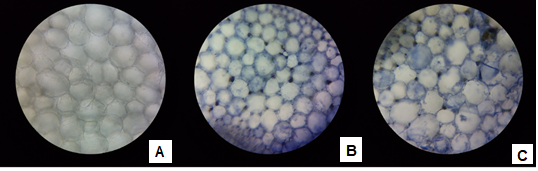


**Figure S6:** Colonization of endophytic fungal isolate A) Control B) Cp1 and C) Cp2 within soybean roots cross-section using lacto-phenol cotton blue staining.
